# Association of TRAF2 with the short form of cellular FLICE-like inhibitory protein prevents TNFR1-mediated apoptosis

**DOI:** 10.1186/1750-2187-3-2

**Published:** 2008-01-14

**Authors:** Dong-Joon Kim, Chan Park, Bermseok Oh, Young-Youl Kim

**Affiliations:** 1 Center for Genome Sciences, National Institute of Health in Korea (KNIH), Nokbun-dong 5, Eunpyung-Gu, Seoul 122-701, Korea

## Abstract

**Background:**

We have previously shown that c-FLIP_L _is a more potent inhibitor than c-FLIP_S _of Fas ligand-induced apoptosis and that c-FLIP_L _physically binds to Daxx, an alternative Fas-signaling adaptor. Here we examined whether c-FLIP_S _effectively inhibits TNFR1-mediated apoptosis and triggers JNK activation through its interaction with TRAF2.

**Results:**

Some cancer cell lines, such as DU145, AGS, and PC3, have higher levels of c-FLIP_S _than other cell lines, such as SNU-719 and T24. The expression of c-FLIP_S _correlated with the susceptibility to TNFR1-mediated apoptosis. In contrast to DU145 and PC3, which are resistant to TNFR1-mediated apoptosis, T24 and SNU719 were sensitive to TNF-α treatment. To address the role of c-FLIP_S _in TNFR1-mediated apoptosis, we examined the molecular interaction between c-FLIP_S _and TRAF2. As expected, western blot analysis revealed that TRAF2 antibody immunoprecipitated a greater amount of c-FLIP_S _than c-FLIP_L_. Also, we measured the involvement of c-FLIP_S _in TNF-α-induced JNK activation and apoptosis by comparing these in TNF-α-resistant and TNF-α-sensitive cell lines. Treatment with TNF-α increased the phosphorylated JNK level in SNU719 and T24 cells, whereas DU145 and AGS cells were resistant to TNF-α-mediated apoptosis.

**Conclusion:**

We now report that the short form of c-FLIP_S _is a more efficient inhibitor of TNF-receptor 1-mediated apoptosis signaling than the long form of the protein.

## Background

Tumor necrosis factor receptor 1 (TNFR1) exhibits diverse activities inducing apoptosis and activating transcription factor NF-κB, which lead to the induction of a number of anti-apoptotic factors. After the components of the death-inducing signaling complex of the related death receptor CD95 (APO-1/Fas) [1] were identified, it was widely assumed that TNFR1 also recruits both the FADD adaptor and caspase 8 upon binding of its ligand, TNF-α, resulting in the subsequent initiation of apoptosis. However, all attempts to demonstrate a direct physical association of FADD and caspase 8 with TNFR1 have so far been unsuccessful [2], whereas the anti-apoptotic components of the signaling complex containing TNF-α receptor-associated factor 2 (TRAF2), TNFR1-associated death domain protein (TRADD) and c-IAP1 are readily detectable [3].

In contrast to our understanding of the FAS and TRAIL receptors, the molecular mechanisms underlying TNFR1-induced cell death remain poorly defined, despite extensive study of the signaling pathways that operate through this receptor [4,5]. It is currently believed that the engagement of TNFR1 triggers the recruitment of the DD-containing adaptor molecule, TRADD, followed by the DD-containing Ser/Thr kinase RIP1 [6]. This signaling complex is required for TRAF2/5 and c-IAP1 binding, which leads to triggering of the NF-κB and JNK signaling pathways [7]. TNFR1 binding by its ligand triggers these pathways, and can induce apoptosis by alternately binding the DD-containing adaptor FADD (through TRADD), which facilitates caspase 8 recruitment and activation [8].

The activation of the TNFR1 death receptor by TNF-α leads to the recruitment of TRADD, which serves as a platform for the formation of various signaling complexes involved in different biological processes [7]. For instance, TRADD can recruit FADD and promote caspase-8 activation and apoptosis through the extrinsic pathway [8]. However, TNF-α is not cytotoxic to most cells because TRADD can also recruit TRAF2 and RIP to form distinct complexes leading to the activation of NF-κB and JNK [8-10]. Because activation of NF-κB serves as a primary mechanism to protect cells against apoptotic stimuli such as TNF-α [11-13], TNF-α-induced apoptosis requires NF-κB inhibition.

The involvement of JNK in TNF-α-mediated apoptosis is highly controversial [14-17]. Induction of NF-κB has been shown recently to inhibit TNF-α-mediated JNK activation. Moreover, blocking NF-κB results in the sustained activation of JNK, which may directly promote TNF-α-mediated apoptosis [14,17]. However, although sustained activation of JNK promotes cell death, the molecular basis of how this contributes to TNF-α-mediated apoptosis remains to be addressed.

One of the well described apoptosis inhibitors is c-FLIP, which is also known as FLAME-1/I-FLICE/CASPER/CASH/MRIT/CLARP/Usurpin [18]. c-FLIP_L _and c-FLIP_S _contain two DED domains that are structurally similar to the N-terminal part of procaspase-8. The C terminus of c-FLIP_L _consists of two catalytically inactive caspase-like domains (p20 and p12), whereas the short C terminus of c-FLIP_S _shows no homology to procaspases-8 or -10. Both isoforms of c-FLIP are recruited to the DISC by DED-DED interactions [19-21]. However, it remains to be clarified in detail whether the differences in the mechanism of apoptosis inhibition also reflect different functional roles of c-FLIP_S _and c-FLIP_L _and by which downstream mechanisms they are mediated.

Genetic evidence suggests that FADD and caspase 8 are important for TNFR1-mediated apoptosis [22,23]. In addition, expression of the inhibitor of caspase 8, c-FLIP_L_, inhibits the TNF-α-induced apoptotic pathway [24], which provides further evidence for the important role of caspase 8 in this cascade. Expression of c-FLIP_L _is induced by NF-κB [25], which may explain why death receptor-induced apoptosis is generally blocked in cells with active NF-κB.

Here we examined whether c-FLIP_S _effectively inhibits TNFR1-mediated apoptosis and triggers JNK activation through its interaction with TRAF2. The effects of TRAF2 in TNFR1-mediated apoptosis was attenuated by DN-TRAF2, and the level of p-JNK in c-FLIP_S _siRNA increased more in TNF-α sensitive cell line than in a TNF-α resistant cell line. In this paper, we demonstrate that the regulation of c-FLIP_S _is related to the TNFR1 signaling pathway.

## Results

### Inhibition of TNFR1-mediated apoptosis is dependent on the expression levels of c-FLIP_S_

**Figure 1:**
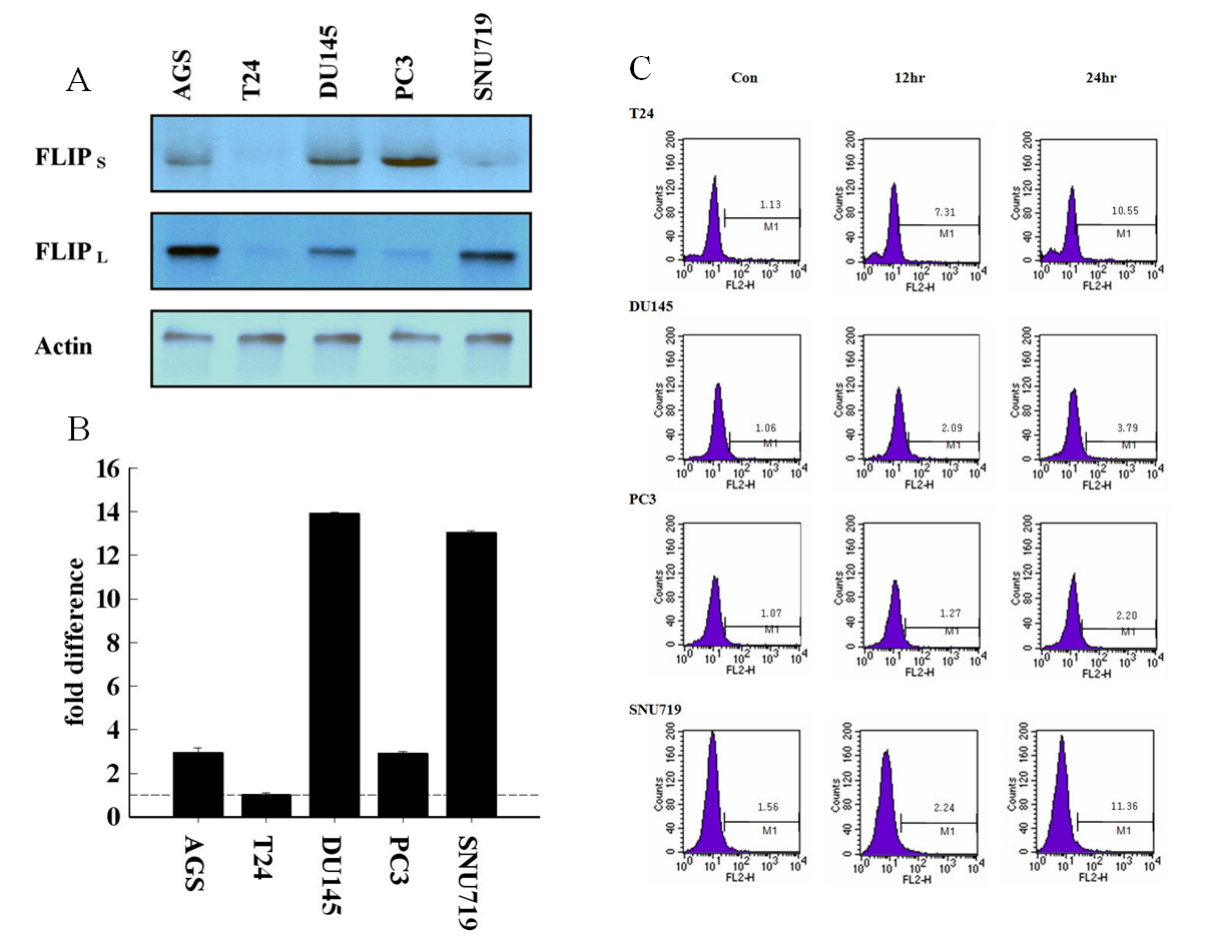
**The expression levels of c-FLIP_S _indicate a relationship to resistance to TNF-induced apoptosis**. (A) Immunoblotting analysis of c-FLIP_L _and c-FLIP_S _in TNF-α-resistant and TNF-α-sensitive cell lines with anti-FLIP_L _and anti-FLIP_S _antibodies. The molecular weights of c-FLIP_L _and c-FLIP_S _were about 55 kDa and 28 kDa, respectively. β-Actin was used as a loading control. (B) Differences in the expression levels of c-FLIP_S _between various cancer cell lines shown by quantitative real-time PCR and SYBR Green detection. Levels of c-FLIP_S _were normalized to GAPDH and the fold changes in c-FLIP_S _levels are shown for each cell line. Data shown are the average of two assays. PCR products were analyzed using Sequence Detection software. Error bars equal the SEM. (C) Measurement of TNF-α-induced apoptosis using the TUNEL assay (M, TUNEL positive) in different human cancer cell lines. Different patterns are shown following treatment with TNF-α (10 ng/ml) at 0, 12, and 24 h.

To examine the relationship between the expression level of c-FLIP_S _and the apoptotic response, we used real-time PCR to examine the expression of c-FLIP in human cancer cell lines. We have shown previously that c-FLIP_L _is a more potent inhibitor than c-FLIP_S _of Fas ligand-induced apoptosis and that c-FLIP_L _physically binds to Daxx through an interaction between its C-terminal domain and the Fas-binding domain of Daxx, an alternative Fas-signaling adaptor [26]. Interestingly, some cancer cell lines, such as DU145, AGS, and PC3, have higher levels of c-FLIP_S _than other cell lines, such as SNU-719 and T24 (Fig. 1A). However, the expression of c-FLIP_S _at the transcriptional level does not exactly reflect the protein levels (Fig. 1B). This discrepancy may result from the low mRNA stability of c-FLIP, which has been described recently [27,28].

To elucidate the biological significance of these phenomena, we examined the susceptibility of these cell lines to TNF-α. Interestingly, the expression of c-FLIP_S _correlated with the susceptibility to TNFR1-mediated apoptosis (Fig. 1C). In contrast to DU145 and PC3, which are resistant to TNFR1-mediated apoptosis, T24 and SNU719 were sensitive to TNF-α treatment.

### The sensitivity of cells to TNF is associated with the expression levels of c-FLIP_S_

**Figure 2:**
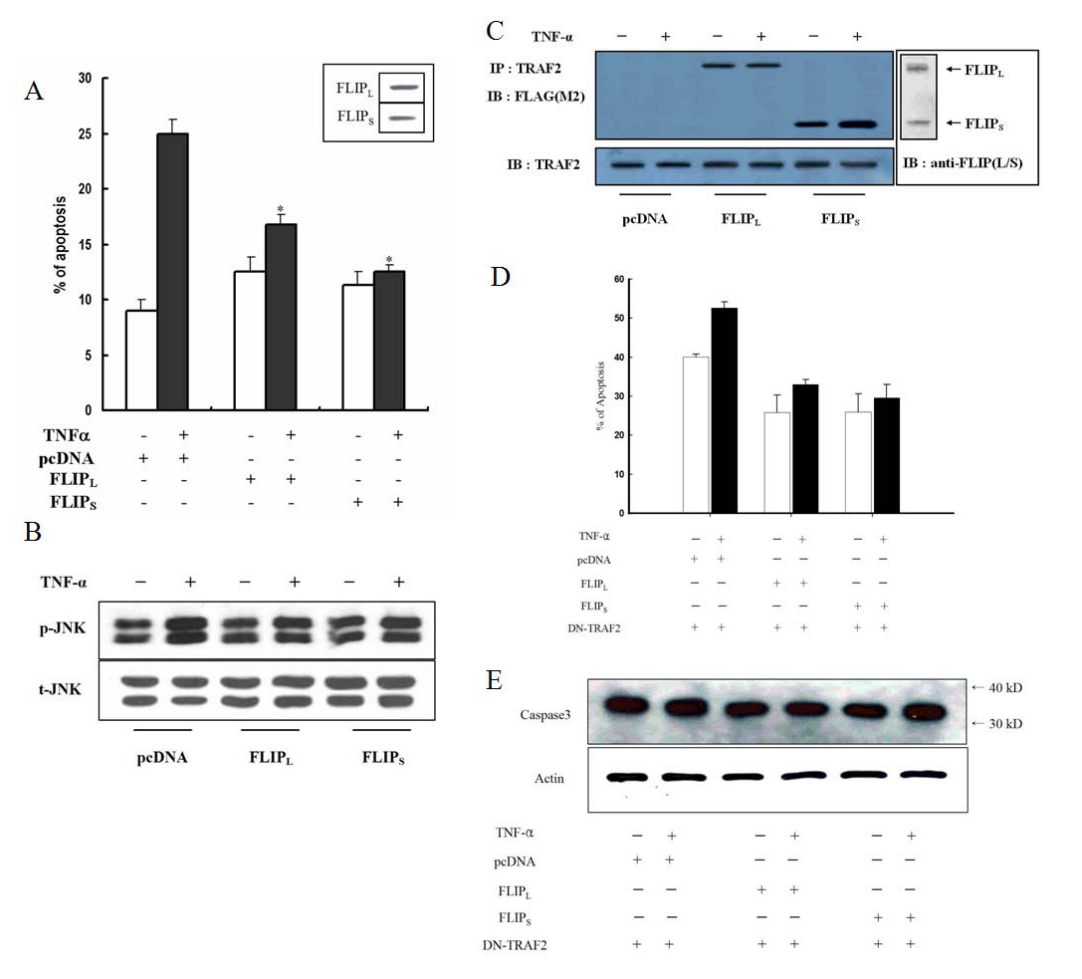
**Expression of c-FLIP_S _confers stronger resistance to TNF-α-induced apoptosis than c-FLIP_L_, through a TRAF2-dependent mechanism**. (A) Analysis of TNF-α-induced apoptosis. SNU-719 cells were transfected with the indicated constructs and treated with 10 ng/ml TNF-α for 24 h. Apoptotic cells were evaluated 48 h after transfection for each construct using the DNA fragmentation assay as described in Materials and methods. After treatment with TNF-α, the percentage of apoptotic cells transfected with pcDNA and c-FLIP_L _increased, but the percentage of apoptotic cells did not change in cells transfected with c-FLIP_S_. This experiment was performed three times independently. *P < 0.01 compared with FLIP_L _and FLIP_S_. (B) Ectopic expression of c-FLIP_L _by itself induced the activation of JNK in response to TNF-α, whereas c-FLIP_S _did not change this effect. P- and t-JNK indicate the phosphorylated JNK and total JNK levels, respectively. (C) SNU-719 cells were transfected with FLAG-tagged c-FLIP_L _and c-FLIP_S _expression vectors, incubated with TNF-α (10 ng/ml) for 12 h, and subjected to immunoprecipitation with anti-TRAF2 antibody followed by immunoblotting with anti-FLAG antibody. Immunoblotting of TRAF2 was used as a loading control. (D) To verify the role of TRAF2 in TNF-α-induced apoptosis, SNU-719 cells were cotransfected with the indicated constructs with DN-TRAF2. Apoptotic cells were evaluated 24 h after transfection for each construct using flow cytometric analysis as described in Materials and methods. (E) To confirm the results, we examined caspase 3 activity in the same conditions as described in Fig.2. D. β-Actin was used as a loading control.

Based on our previous findings, we speculated that the overexpression of c-FLIP_S _would confer resistance to TNF-α-induced apoptosis more effectively than overexpression of c-FLIP_L_. We investigated the role of c-FLIP_S _in this pathway by transfecting into SNU-719 cells, which express a lower level of c-FLIP_S _than other cell lines, because we did not have a knockout cell line of c-FLIP_S_. Although the ectopic expression of c-FLIP_S _and c-FLIP_L _did not significantly inhibit TNF-α-induced apoptosis, the inhibition of TNFR1-induced apoptosis by c-FLIP_S _was more potent than that of c-FLIP_L _(Fig. 2A).

To further examine the role of c-FLIP_S _in JNK activation, we transfected c-FLIP_L _and c-FLIP_S _expression vectors into SNU-719 cells, which constitutively express TNF. We found other significant differences in the effects of these c-FLIP variants on the TNF-α-induced activation of JNK. In c-FLIP_L_-transfected cells, TNF induced the phosphorylation of JNK, whereas in c-FLIP_S_-expressing cells, the phosphorylation of JNK was not changed (Fig. 2B). To address the role of c-FLIP_S _in the activation of JNK, we examined the molecular interaction between c-FLIP_S _and TRAF2, a mediator of TNF-induced JNK and NF-κB activation. Extracts from cell lines expressing pcDNA, c-FLIP_L_, and c-FLIP_S _were immunoprecipitated with TRAF2 or with the corresponding preimmune serum. As expected, western blot analysis revealed that TRAF2 antibody immunoprecipitated a greater amount of c-FLIP_S _than c-FLIP_L _(Fig. 2C). To verify whether TRAF2 mediates TNFR1-induced apoptosis, DN-TRAF2 was transiently expressed using the same conditions as shown in Fig. 2A (Fig. 2D). The effects on TNF-α-induced apoptosis did not differ between the c-FLIP variants. To measure the activity of apoptosis, we also examined the level of caspase 3 by immunoblotting after treatment with DN-TRAF2. As expected, the level of caspase 3 protein did not differ between the three c-FLIP variants (procaspase data not shown) (Fig. 2E). These results suggest that each c-FLIP variant is capable of binding to TRAF2, where it may inhibit TNFR1-mediated apoptosis.

### The function of c-FLIP_S _is exerted through TNFR1-mediated JNK activation

**Figure 3:**
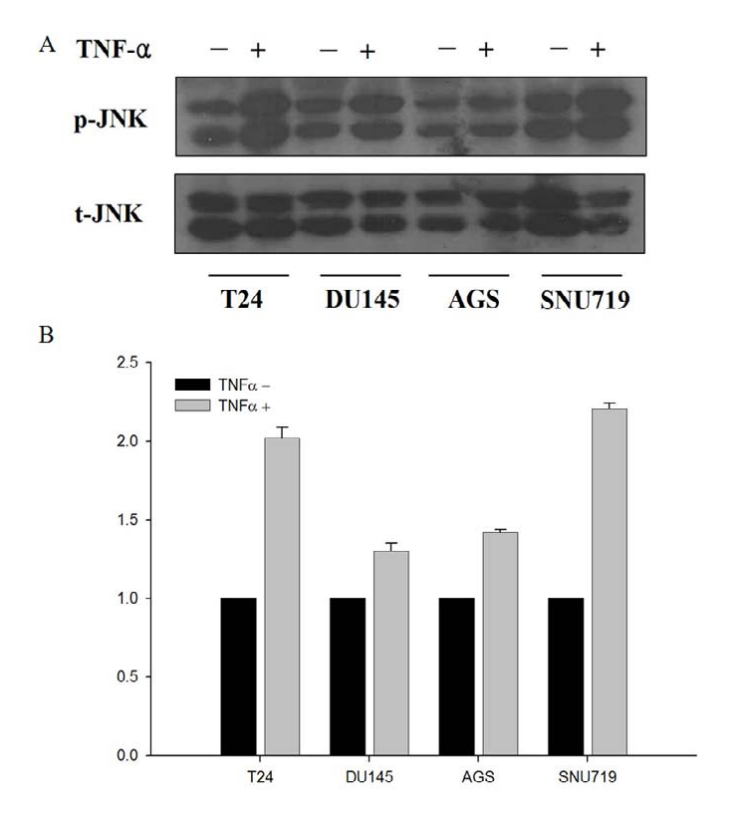
**JNK activation in TNF-resistant cell lines**. TNF-α-sensitive cell lines can activate JNK in response to TNF-α. In T24 and SNU719 cells, JNK is activated in response to TNF-α, whereas DU145 and AGS cells do not show this induction. (A) To examine the activation of JNK, protein extracts from the indicated cell lines were incubated for 6 h with TNF-α (10 ng/ml) and subjected to immunoblotting with phospho-specific and total JNK antibodies. P- and T- indicate phosphorylated proteins and total proteins, respectively. (B) Expression was quantified by densitometric scan of the blots (A). Values are the ratio of p-JNK and t-JNK and are presented relative to each control.

The TNFR1-mediated apoptotic response has two pathways: type 1 apoptosis achieved through FADD-caspase 8 activation, and type 2 mitochondrial-dependent apoptosis, which is less-dependent on the FADD-caspase 8 pathway [28]. TNF-α enhances the activity of JNK, which leads to dysfunctional mitochondria and contributes to TNF-α-induced apoptosis [29]. We examined the involvement of c-FLIP_S _in TNF-α-induced JNK activation and apoptosis by comparing these in TNF-α-resistant and TNF-α-sensitive cell lines. Treatment with TNF-α increased the phosphorylated JNK level in SNU719 and T24 cells, whereas DU145 and AGS cells were resistant to TNF-α-mediated apoptosis (Fig. 3A and 3B). These results indicate that the sensitivity to TNF-α is associated with the TNF-induced activation of JNK.

### Knockdown of endogenous c-FLIP_S _augments TNFR1-mediated apoptosis

**Figure 4:**
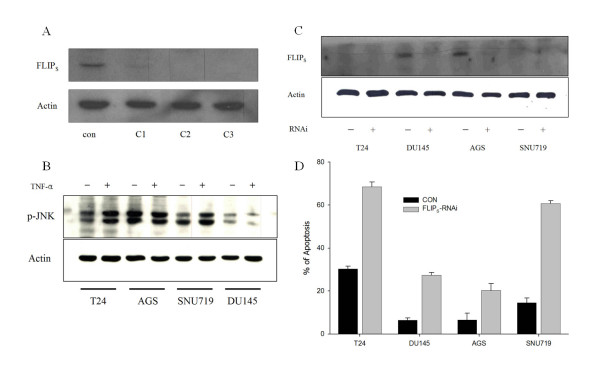
**Knockdown of endogenous c-FLIP_S _in several cell lines enhances apoptosis induction by TNF-α**. (A) To examine the role of c-FLIP_S _in TNFR1-mediated apoptosis, PC3 cells were transfected with three different siRNA oligonucleotides designed to knock down c-FLIP_S_. The level of c-FLIP_S _protein was inhibited completely by C2 and C3. In further studies, we used C3 oligonucleotide as an siRNA. (B) The indicated siRNA-transfected cells were treated with TNF-α (5 ng/ml) for 24 h and extracted for immunoblotting with phospho-specific antibodies to JNK and actin. (C) The indicated siRNA-transfected cell lines were treated with TNF-α (5 ng/ml) for 24 h before harvesting for flow cytometric analysis. This experiment was performed independently three times. (D) The indicated siRNA-transfected cell lines were treated with TNF-α (5 ng/ml) for 24 h and extracted for immunoblotting with anti-FLIP_S _antibody. β-Actin was used as a loading control.

To confirm the involvement of endogenous c-FLIP_S _in TNFR1-mediated apoptosis, we developed siRNA oligonucleotides that selectively knock down the expression of c-FLIP_S _in several cancer cell lines. To select a potent candidate siRNA oligonucleotide, we transfected human PC3 cells, which express a higher level of c-FLIP_S _than other cell lines (Fig. 1A), with siRNAs. Cells were incubated for 48 h, and the level of c-FLIP_S _proteins was assessed by immunoblot analysis (Fig. 4A). The results confirmed the ability of oligonucleotides C2 and C3 to knock down expression of c-FLIP_S_. To verify the effect of c-FLIP_S _knockdown on JNK activation, we studied several cancer cell lines (Fig. 4B). Phosphorylated JNK levels increased in T24 and SNU719 cells, whereas AGS and DU145 cells were resistant to TNF-α. We also examined the susceptibility of these cell lines to TNF-α in c-FLIP_S _siRNA (Fig. 4C). The basal level of c-FLIP_S _was marginal in T24 and SNU719 cells, but DU145 and AGS cell lines expressed some degree of c-FLIP_S _protein in the control condition (Fig. 1A). Relative to the basal level of c-FLIP_S_, the ratio of apoptosis increased more in DU145 (7 fold) and AGS cells (6 fold) than in T24 cells (2.5 fold), although these differences were not considerable. Apoptosis analysis indicated that knockdown of c-FLIP_S _in SNU719 and T24 cells increased the level of spontaneous cell death by about 40% compared with control, whereas knockdown of DU145 and AGS had minimal effects. To confirm this result, we used immunoblot analysis to examine the level of c-FLIP_S _protein in these cell lines after treatment with siRNA (Fig. 4D). As expected, the expression of c-FLIP_S _decreased in these cell lines with c-FLIP_S _siRNA.

## Discussion

Although a specific role for each c-FLIP and a distinct mechanism by which each inhibits apoptosis have been proposed [30,31], the functions of the c-FLIP splice variants remain largely unknown. Our current data provide insight into the mechanism underlying the TNF-α-induced apoptotic pathway. This pathway is characterized by a higher affinity of TRAF2 for c-FLIP_S _over c-FLIP_L _and involves suppression of JNK activation and resistance to TNF-α-induced cell death. c-FLIP_S _is also readily detectable in various types of human cancer and confers resistance to CD95-mediated apoptosis in T cells during the immune response [32].

**Figure 5:**
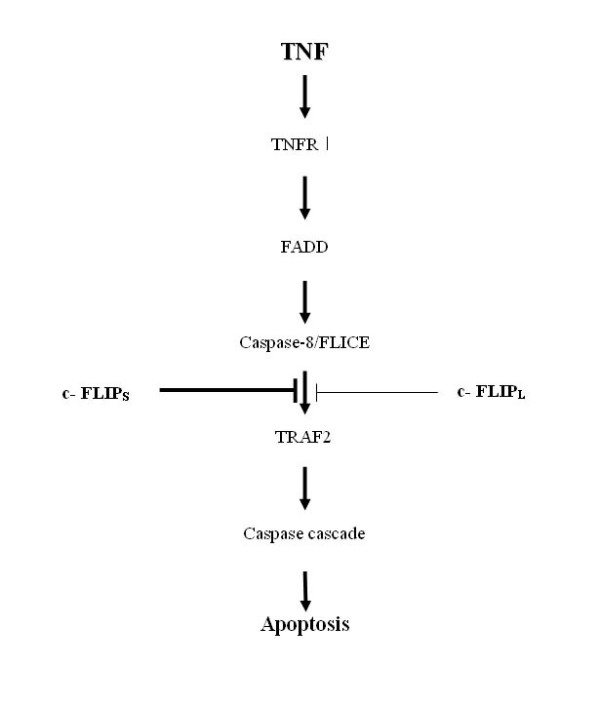
**A working model of the role of c-FLIP_S _in TNFR1-mediated apoptosis**. The binding of c-FLIP_S _to TRAF2 delays the recruitment of c-FLIP_L_, and c-FLIP_S _and c-FLIP_L _block TNFR1-mediated apoptosis. However, the cellular c-FLIP_S _level is elevated sufficiently to block apoptosis and promote cell survival.

Our results are summarized in the model shown in Fig. 5. It appears that c-FLIP_S _and c-FLIP_L _have distinct roles, and we speculate that c-FLIP_S _acts primarily to support TNF-induced JNK activation. Our results are consistent with previous data showing different functions of each type of c-FLIP, including a specific role for c-FLIP_S _in CD40 signaling [33]. A distinct action of each c-FLIP variant in Fas-induced cleavage of caspase 8 has also been proposed [34]. According to these earlier studies, c-FLIP_L _and c-FLIP_S _produce different end products in the caspase 8 pathway. Our data also show that apoptosis sensitivity correlates with c-FLIP expression in several cell types, including dendritic cells, keratinocytes, and vascular smooth muscle cells (data not shown).

Although TRAF2 bound both c-FLIP_S _and c-FLIP_L _in our experiments, other reports have shown that c-FLIP_L _interacts exclusively with TRAF2 [35]. This discrepancy may be reconciled by recent studies showing that alternative variants of c-FLIP promote different TNF-α-induced signaling pathways. c-FLIP_L _has a strong affinity for Raf, whereas c-FLIP_S _is tightly associated with TRAF2 [30].

The gene knockout and siRNA knockdown studies of c-FLIP reported to date show that reduction of both c-FLIP variants sensitizes cells to death receptor-induced apoptosis [36]. We used a selective siRNA knockdown approach to assess the specific function of endogenous c-FLIP_S _and compare this with the function of c-FLIP_L_. Our results reinforce the conclusion that endogenous c-FLIP_S _inhibits death receptor-mediated apoptosis.

Our data showing that c-FLIP_S _interacts rapidly with FADD and enhances the activation of JNK through TRAF2 may explain why c-FLIP_S _shows high expression levels, despite its overlapping role with c-FLIP_L_. In addition, the expression pattern of c-FLIP_S _is coupled to the expression of TNF. Our current experiments have confirmed our previous data showing that each type of c-FLIP has a distinct target in the inhibition of apoptosis. We postulate that the different expression patterns of the c-FLIP variants are specific to cancer cell type.

## Methods

### Cell culture and reagents

Human cancer cell lines were obtained from ATCC and maintained in RPMI 1640 containing 10% fetal bovine serum (FBS) (Gibco BRL) heat inactivated at 37°C; cells were grown in a humidified chamber containing 5% CO_2_. Except as indicated, all other chemicals used in this experiment were purchased from Sigma-Aldrich. Recombinant human TNF-α and antibodies were obtained from Santa Cruz Biotech.

### Construction of vectors and transfection procedure

Expression constructs for c-FLIP_L_, c-FLIP_S_, and a dominant-negative version of TRAF2 (TRAF2^266–501^) were generated by RT-PCR-based cloning into the pcDNA 3.1 topo His/V5 vector (Invitrogen.) Geneporter transfection reagent was purchased from Gene Therapy Systems. The transfection procedure was performed according to the manufacturer's instructions. Briefly, cells were seeded in six-well plates at a concentration of 1 × 10^5 ^cells/well. Four micrograms of DNA per well was mixed with serum-free media containing Geneporter (10 μl/well), incubated for 30 min at room temperature, and added to cells that had previously been washed twice with PBS. After 3–5 h, media (1 ml/well) containing 20% FBS was added to each well. All experiments, including thymidine-incorporation assays, western blotting, immunoprecipitation, and cell death assays, were performed after incubation for 24 h. To monitor transfection efficiency, cells were cotransfected with GFP-pcDNA.

### siRNA preparation and transfection

Small interfering RNA (siRNA) oligonucleotides against c-FLIP_S _were synthesized by BLOCK-iT™ RNAi Designer [37] (C1: 5'GCCATTTGACCTGCTCAAA3' C2: 5'GCAGAGATTGGTGAGGATT3' C3: 5'GGAGAAACTAAATCTGGTT3'). All siRNA oligonucleotides were resuspended to a concentration of 20 μM. The cells were seeded for transfection in medium without antibiotics one day before transfection to ensure they were 85%–95% confluent on the day of transfection. For transfection, regular medium was replaced with serum-free medium without antibiotics. The cells were transfected with siRNA using Lipofectamine 2000 (Invitrogen) at a ratio of 1:3 siRNA:Lipofectamine (μg:μl), giving a final concentration of 100–150 nM siRNA. The cells were incubated with the siRNA-Lipofectamine 2000 complex for 4 h, the serum-free medium was replaced with normal medium (containing 10% FBS) without antibiotics, and the cells incubated for a total of 48 h before further analysis.

### Real-time PCR

Reactions for real-time PCR were performed in a final volume of 25 μl using TaqMan detection. Each tube contained 12.5 μl of TaqMan Universal PCR Master Mix, 1.25 μl of Assays-on-Demand Gene Expression probes (Applied Biosystems) TaqMan probe and 100 ng of each cDNA template. All PCR samples and controls were prepared in triplicate using 0.2 ml Micro-Amp Optical reaction tubes and MicroAmp Optical tube caps (Applied Biosystems). The PCR mixture was held at 50°C for 5 min and denatured at 95°C for 10 min. Forty amplification cycles were carried out at 95°C for 20 s, followed by 60°C for 1 min. All amplifications were performed using an ABI PRISM^® ^7900 HT (Applied Biosystems). PCR products were analyzed using Sequence Detection software (Applied Biosystems).

### Cell death assay and flow cytometric detection of apoptosis by annexin V labeling

A DNA fragmentation assay (BMS; Manheim, Germany) was used. All procedures were performed using the manufacturer's protocol. Analysis of ELISA was carried by a Softmax ELISA reader at an absorbance of 460 nm. Each experiment was performed independently three times by external observers.

Annexin V (Sigma), a phosphatidylserine-binding protein, was used to detect apoptotic cells, and the nuclear stain, propidium iodide (PI) (Sigma) was used to identify late-apoptotic cells or necrotic cells. Cells seeded at a concentration of 1 × 10^5 ^cells/well in six-well plates were incubated at 37°C with or without 10 ng/ml TNF-α for 24 h. Cells were washed twice with cold PBS and resuspended in 500 μl of binding buffer (100 μM HEPES, pH 7.4, 14 mM NaCl, and 25 μM CaCl_2_) containing 5 μl of annexin V-fluorescein isothiocyanate (FITC) (Sigma) and 10 μg/ml PI for 15 min at 4°C in the dark. For each sample, about 10^5 ^cells were analyzed by flow cytometry. FITC and PI emissions were collected through 520 and 630 nm bandpass filters, respectively.

## Authors' contributions

DJK did all of the experiments and writing of the manuscript,

CP and BSO contributed experiment design and discussion of results.

YYK contributed the project leader and designer of this study and writing of the manuscript.

All authors read and approved the final manuscript.
